# Mortality Trends in Heart Failure and Pneumonia Among US Adults Aged 65 and Older: Analysis of CDC WONDER Data

**DOI:** 10.7759/cureus.91162

**Published:** 2025-08-28

**Authors:** Faraz Ahmad, Ans Ahmad, Labiba Mansoor, Muhammad Kamran Khan, Mansoor Ahmad

**Affiliations:** 1 Department of Cardiology, Tahir Heart Institute, Chenab Nagar, PAK; 2 Department of Cardiology, Meritus Medical Center, Hagerstown, USA

**Keywords:** covid-19, elderly population, epidemiology, heart failure, mortality trend, pneumonia, racial and ethnic disparities

## Abstract

Background: Heart failure (HF) and pneumonia are leading causes of morbidity and mortality in older adults. Their coexistence poses major clinical challenges and complicates management. This study quantified and analyzed mortality trends where both HF and pneumonia were listed as multiple causes of death, with attention to disparities by sex, race/ethnicity, age, region, state, and urban-rural status.

Methods: We conducted a retrospective observational analysis using the CDC WONDER multiple-cause mortality database (1999-2020), including deaths in which both HF and pneumonia were recorded on death certificates. Age-adjusted mortality rates (AAMRs) per 100,000, with 95% confidence intervals (CIs), were calculated across demographic, geographic, and temporal variables using ICD-10 codes. Joinpoint regression identified statistically significant (p < 0.05) trend changes and annual percent changes (APCs).

Results: A total of 502,834 deaths occurred in adults ≥65 years with both HF and pneumonia. In men, AAMR declined from 109.4 in 1999 to 63.5 in 2020; in women, from 80.3 to 40.7. Non-Hispanic (NH) White adults had the highest racial AAMR (56.7). The Midwest reported the highest regional AAMR (100.4), while West Virginia ranked highest among states (88.0). Non-metropolitan areas showed a higher AAMR (72.9) compared with metropolitan areas.

Conclusions: From 1999 to 2018, HF and pneumonia mortality rates declined but rose again in 2020, potentially reflecting pandemic-related impacts and increased vulnerability in this population. Persistent disparities were observed across sex, race/ethnicity, geography, and urban-rural status. These findings underscore the need for targeted public health strategies to reduce mortality and narrow disparities in older adults with HF and pneumonia.

## Introduction

Heart failure (HF) and pneumonia are major contributors to morbidity and mortality among adults aged ≥65 years [1]. In the United States, an estimated 6.7 million individuals aged ≥20 are affected by HF [2], while pneumonia accounts for around 1.4 million emergency department visits annually [3]. Their coexistence poses significant clinical challenges in older adults due to comorbidities and age-related physiological vulnerability [4]. Patients with HF are particularly susceptible to respiratory infections, with pneumonia contributing to over 1.5 million hospitalizations each year [4]. Infection can precipitate acute HF decompensation, further compromise cardiovascular function, and markedly increase mortality risk [5]. This vulnerability is attributed to impaired pulmonary function, systemic inflammation, and reduced immune response inherent to HF [6].

Studies indicate that patients with HF have nearly double the risk of developing pneumonia, with worse recovery outcomes and higher hospitalization rates [4,7]. The coexistence of these conditions is consistently associated with adverse outcomes, including excess mortality in older adults [4].

While HF and pneumonia mortality have been examined independently, national-level data describing their combined impact remain limited. To address this gap, we used the CDC WONDER multiple-cause mortality database to analyze trends in deaths listing both HF and pneumonia, focusing on disparities by age, sex, race/ethnicity, and geography. These findings provide critical insights to inform targeted public health strategies for this high-risk population.

## Materials and methods

Study design and population

We conducted an observational analysis using death records from the CDC WONDER Multiple Cause of Death database for the period 1999-2020 [8], focusing on individuals aged ≥65 years in the United States. Mortality records were reviewed for cases in which both heart failure (HF) and pneumonia were listed among the causes of death. The 10th edition of the International Classification of Diseases (ICD-10) was used to identify relevant cases. HF was classified under I50 codes, including congestive HF (I50.0), left ventricular failure (I50.1), and unspecified HF (I50.9). Pneumonia was identified using J12-J18 codes, encompassing viral (J12), bacterial (J13-J15), chlamydial and other specified types (J16), and unspecified cases (J18) [9]. Deaths were included only if both HF and pneumonia codes appeared; those without both codes were excluded. Because this study used publicly available, de-identified government data, Institutional Review Board approval was not required.

Data abstraction

The CDC WONDER dataset includes information on sex, race/ethnicity, urban-rural classification, region, state, age group, and place of death [8]. Sex was classified as male or female based on death certificate records. Race/ethnicity followed the Office of Management and Budget (1997) standards: Hispanic or Latino, non-Hispanic (NH) White, NH Black or African American, NH American Indian or Alaska Native, and NH Asian or Pacific Islander. Urban-rural status was defined according to the National Center for Health Statistics (2013) scheme, which classifies urban areas as large metropolitan regions (≥1 million population) or medium/small metropolitan regions (50,000-999,999 population). Rural areas are defined as non-metropolitan regions with <50,000 population. Geographic regions were categorized by the US Census Bureau (2013) as West, Midwest, South, or Northeast. Age groups were stratified as 65-74, 75-84, and ≥85 years. Place of death was grouped into healthcare settings (including outpatient, emergency department, inpatient, dead on arrival, or unknown status), home, hospice/palliative care, and nursing/extended care facilities. Data covered all 50 states and Washington, D.C. The mortality variables available through CDC WONDER have been widely used in prior epidemiologic studies and form the basis of the present analysis.

Statistical analysis

National trends were evaluated using age-adjusted mortality rates (AAMRs) per 100,000 individuals for HF and pneumonia, adjusted for population age structure using the 2000 U.S. standard population as the reference. Mortality rates were analyzed for 1999-2020 by sex, race/ethnicity, age group, urban-rural status, state, and year. Temporal patterns in AAMRs were assessed using the Joinpoint Regression Program (Version 5.3.0.0) [10], which applies log-linear regression models to identify statistically significant changes in trends. Annual percent changes (APCs) with 95% confidence intervals (CIs) were calculated, and trends were classified as increasing or decreasing when the slope significantly differed from zero. A p-value <0.05 was considered statistically significant.

## Results

A total of 502,834 deaths involving both HF and pneumonia were recorded among adults aged ≥65 years in the United States between 1999 and 2020 (Table 1).

**Table 1:** Deaths from heart failure and pneumonia stratified by sex and race in older adults in the United States (1999-2020) NH: non-Hispanic.

	Deaths								
Year	Overall	Women	Men	NH White	NH Black or African American	NH Asian or Pacific Islander	NH American Indian or Alaska Native	Hispanic or Latino	Population
1999	31,074	18,168	12,906	27,682	1978	350	85	895	34,797,841
2000	29,662	17,312	12,350	26,383	1874	329	90	910	34,991,753
2001	28,766	16,737	12,029	25,536	1772	355	77	953	35,290,291
2002	28,984	16,700	12,284	25,722	1759	390	88	944	35,522,207
2003	28,761	16,548	12,213	25,577	1689	388	116	934	35,863,529
2004	27,596	15,737	11,859	24,444	1641	417	88	960	36,203,319
2005	28,611	16,366	12,245	25,282	1749	439	115	995	36,649,798
2006	25,856	14,659	11,197	22,698	1545	483	108	983	37,164,107
2007	24,386	13,766	10,620	21,368	1499	441	100	963	37,825,711
2008	20,463	11,849	8614	17,792	1317	382	83	851	38,777,621
2009	18,934	10,679	8255	16,331	1257	404	83	842	39,623,175
2010	18,668	10,426	8242	16,137	1245	380	97	783	40,267,984
2011	19,335	10,805	8530	16,691	1198	425	85	916	41,394,141
2012	17,924	9772	8152	15,323	1182	415	78	891	43,145,356
2013	18,661	10,343	8318	15,865	1228	482	83	968	44,704,074
2014	17,758	9657	8101	15,175	1163	408	95	873	46,243,211
2015	18,851	10,236	8615	16,010	1250	449	93	979	47,760,852
2016	17,712	9288	8424	14,926	1259	434	88	955	49,244,195
2017	18,223	9567	8656	15,252	1247	525	96	1063	50,858,679
2018	18,281	9455	8826	15,213	1364	494	106	1075	52,431,193
2019	17,876	9126	8750	14,745	1373	508	94	1127	54,058,263
2020	26,452	12,770	13,682	20,421	2782	750	164	2291	55,659,365
Total	502,834	279,966	222,868	434,573	33,371	9,648	2112	22,151	928,476,665

Among the 480,877 deaths with available location data, 66.1% occurred in medical settings, 23.2% in nursing homes or extended care services, 6.2% at home, and 2.7% in hospices.

Annual variations

**Figure 1:**
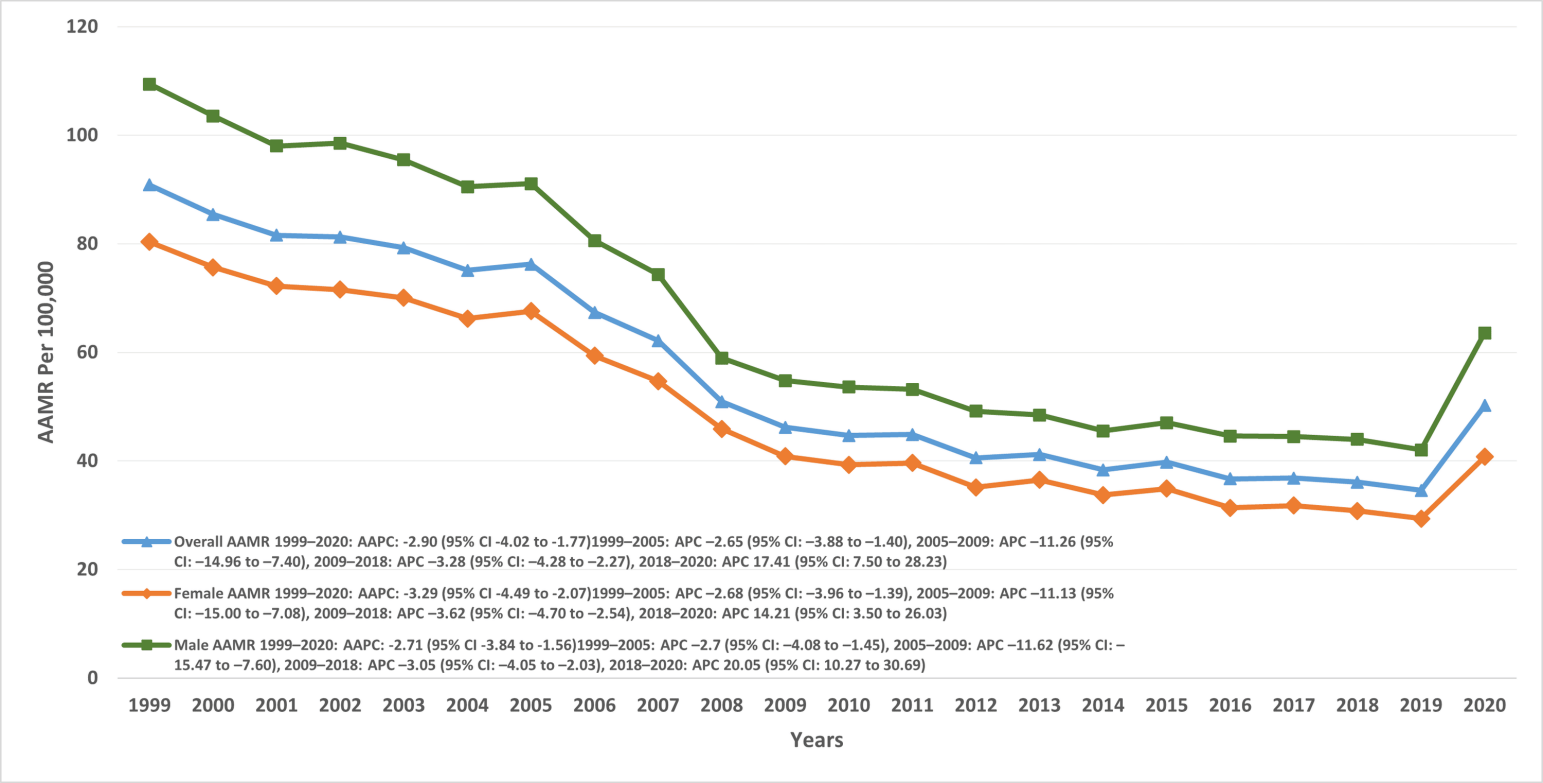
Overall and sex-specific trends in AAMRs for heart failure and pneumonia AAMRs: age-adjusted mortality rates, AAPC: average annual percentage change, APC: annual percent change.

From 1999 to 2020, the AAMR per 100,000 older adults for HF and pneumonia declined from 90.9 to 50.2. The decline was modest between 1999 and 2005 (APC: -2.65; 95% CI: -3.8 to -1.4), steepened between 2005 and 2009 (APC: -11.26; 95% CI: -14.9 to -7.4), and slowed again from 2009 to 2018 (APC: -3.28; 95% CI: -4.2 to -2.2). A marked reversal occurred between 2018 and 2020, with rates rising (APC: 17.41; 95% CI: 7.5-28.2) (Figure 1, Table 2).

**Table 2:** Trends in heart failure and pneumonia mortality among older adults in the United States (1999-2020, AAPC values) and notable Joinpoint segments (2018-2020, APC values) AAPC: average annual percent change, APC: annual percent change, NH: non-Hispanic.

Subgroup	AAPC (1999-2020)	AAPC (95% CI)	AAPC (p-value)	APC (2018-2020)	APC (95% CI)	APC (p-value)
Overall	-2.90	-4.02 to -1.77	<0.001	+17.41	7.50-28.23	0.0021
Female	-3.29	-4.49 to -2.07	<0.001	+14.21	3.50-26.03	0.0128
Male	-2.71	-3.84 to -1.56	<0.001	+20.05	10.27-30.69	0.0006
Northeast	-2.80	-4.17 to -1.42	<0.001	+14.37	2.43-27.70	0.0214
Midwest	-2.76	-4.34 to -1.15	0.001	+19.39	5.10-35.62	0.0108
South	-2.74	-3.88 to -1.58	<0.001	+18.05	8.25-28.74	0.0015
West	-3.54	-5.28 to -1.76	<0.001	+16.40	3.57-30.80	0.0154
NH American Indian/Alaska Native	-2.97	-6.35 to 0.53	0.095	+16.60	-20.33 to 70.63	0.407
NH Asian/Pacific Islander	-3.67	-5.56 to -1.75	0.0002	+15.66	-6.34 to 42.83	0.164
NH Black/African American	-1.52	-2.92 to -0.10	0.036	+44.02	23.38-68.11	0.0001
NH White	-3.03	-4.22 to -1.84	<0.001	+13.82	3.47-25.21	0.0123
Hispanic/Latino	-1.78	-3.15 to -0.38	0.013	+44.03	23.91-67.42	0.00009
Metropolitan	-2.79	-3.98 to -1.58	<0.001	+18.28	7.70-29.90	0.0023
Non-Metropolitan	-3.04	-4.20 to -1.87	<0.001	+14.63	4.24-26.05	0.0090
65-74 years	-1.03	-2.48 to 0.44	0.168	+30.81	21.37-40.99	0.000007
75-84 years	-2.54	-3.83 to -1.23	<0.001	+22.89	11.03-36.01	0.0009
85+ years	-3.66	-4.83 to -2.48	<0.001	+9.92	-0.13 to 20.98	0.053

Disparities by gender

Older men and women demonstrated comparable temporal patterns, but men consistently had higher AAMRs. The overall AAMR was 64.17 per 100,000 in men (95% CI: 63.9-64.4) and 47.8 per 100,000 in women (95% CI: 47.7-48.06), yielding an absolute difference of 16.37 per 100,000. Men had a 34% higher mortality rate than women (RR: 1.34; 95% CI: 1.33-1.35; p < 0.001). In men, the AAMR declined from 109 in 1999 to 91 in 2005 (APC: -2.77; 95% CI: -4.07 to -1.4), followed by a steeper decline to 54.8 in 2009 (APC: -11.62; 95% CI: -15 to -7.6). The downward trend persisted through 2018 (43.9; APC: -3.04; 95% CI: -4.04 to -2.03). From 2018 to 2020, however, a sharp increase was observed (62; APC: 20; 95% CI: 10-30) (Figure 1).

In women, the AAMR decreased from 67.6 in 1999 to 40.8 in 2009, with an initial modest decline from 1999 to 2005 (APC: -2.68; 95% CI: -3.9 to -1.3) followed by a steeper decline from 2005 to 2009 (APC: -11.13; 95% CI: -15 to -7.08). The downward trend continued until 2018 (30.1; APC: -3.62; 95% CI: -4.6 to -2.5). Between 2018 and 2020, mortality again increased (40; APC: 14.2; 95% CI: 3.5-26) (Figure 1, Table 2).

Racial/ethnic disparities

Analysis of AAMRs by race and ethnicity showed the highest mortality in NH White individuals, followed by NH American Indian/Alaska Native, NH Black/African American, Hispanic/Latino, and NH Asian/Pacific Islander populations. The overall AAMRs (per 100,000) were as follows: NH White, 56.7 (95% CI: 56.6-56.9); NH American Indian/Alaska Native, 54.4 (95% CI: 52-56); NH Black/African American, 45 (95% CI: 45.04-46.03); Hispanic/Latino, 38.8 (95% CI: 38.3-39.3); and NH Asian/Pacific Islander, 31.6 (95% CI: 31-32.2).

**Figure 2:**
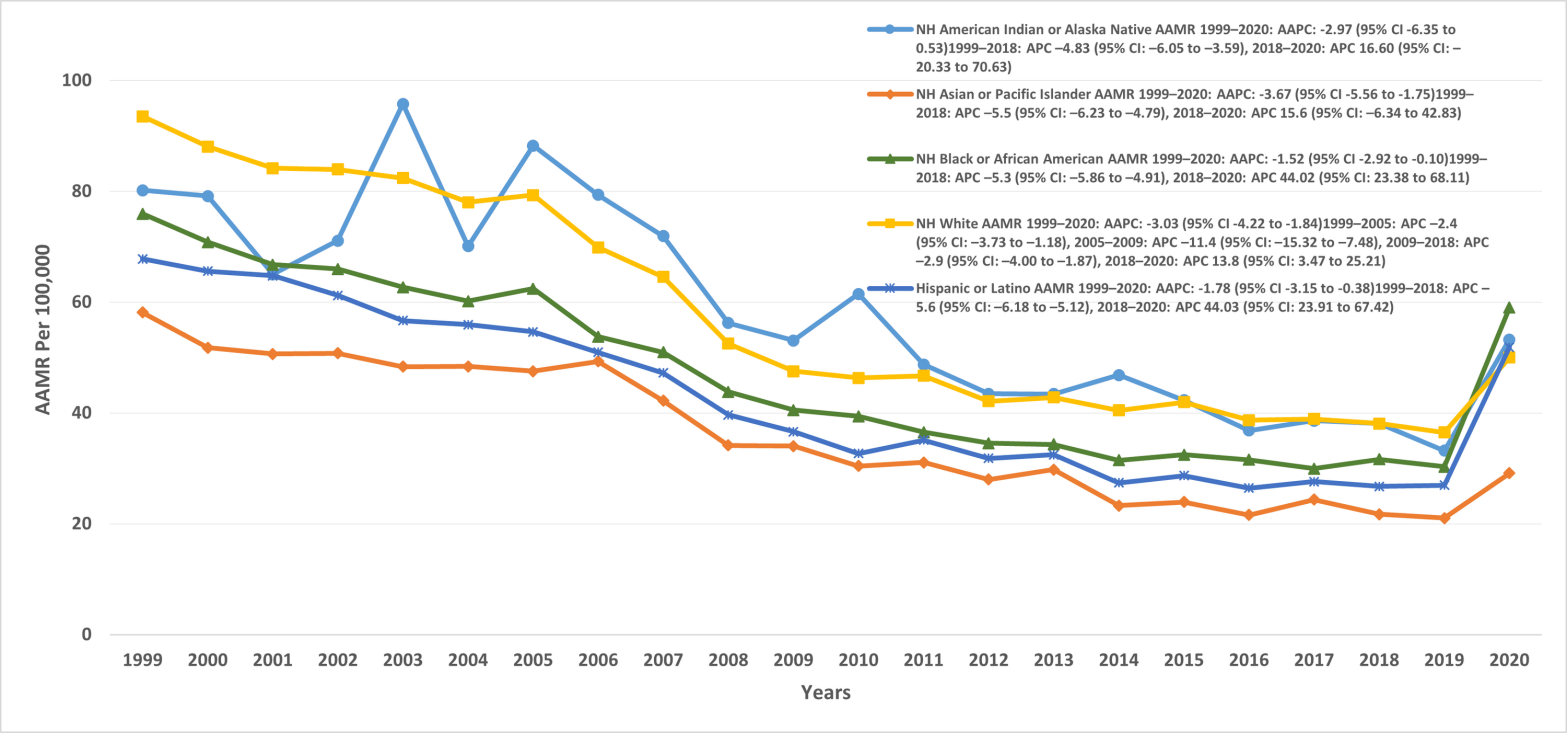
AAMR trends for heart failure and pneumonia stratified by race/ethnicity NH: non-Hispanic, AAMR: age-adjusted mortality rate, AAPC: average annual percentage change, APC: annual percent change.

From 1999 to 2018, all racial/ethnic groups experienced significant declines in AAMR. The steepest reductions were observed in Hispanic/Latino (APC: -5.6; 95% CI: -6.1 to -5.1), NH Asian/Pacific Islander (APC: -5.5; 95% CI: -6.2 to -4.7), NH Black/African American (APC: -5.3; 95% CI: -5.8 to -4.9), and NH American Indian/Alaska Native (APC: -4.8; 95% CI: -6 to -3.5) populations. The NH White population also declined during this period, though in segmented phases: 1999-2005 (APC: -2.46; 95% CI: -3.7 to -1.1), 2005-2009 (APC: -11.4; 95% CI: -15.3 to -7.4), and 2009-2018 (APC: -2.94; 95% CI: -4 to -1.8). Between 2018 and 2020, however, sharp increases emerged across groups. The most pronounced rises were seen in NH Black/African American (APC: 44; 95% CI: 23.3-68) and Hispanic/Latino (APC: 44; 95% CI: 23.9-67.4) populations, followed by NH White (APC: 13.8; 95% CI: 3.4-25.2), NH American Indian/Alaska Native (APC: 16; 95% CI: -20.3 to 70.6), and NH Asian/Pacific Islander (APC: 15.6; 95% CI: -6.3 to 42.8) (Figure 2, Table 2).

Disparities by age groups

The period from 1999 to 2020 revealed distinct trends across age groups (65-74, 75-84, and ≥85 years). For individuals aged 65-74 years, the overall crude mortality rate (CMR) was 11.87 (95% CI: 11.78-11.97). Mortality declined from 1999 to 2006 (APC: -2.5; 95% CI: -3.5 to -1.4), showed a sharper downturn from 2006 to 2009 (APC: -17.1; 95% CI: -24.7 to -8.7), and remained relatively stable through 2018 (APC: -0.15; 95% CI: -1.1 to 0.9). A sharp increase was observed between 2018 and 2020 (APC: 30.8; 95% CI: 21.3-40.9). For the 75-84 years group, the overall CMR was 53.73 (95% CI: 53.47-54). Rates declined between 1999 and 2005 (APC: -2.4; 95% CI: -3.7 to -1.03), dropped more steeply from 2005 to 2009 (APC: -11.5; 95% CI: -15.8 to -7.1), and continued a slower decline until 2018 (APC: -3.4; 95% CI: -4.6 to -2.2). A considerable surge was noted from 2018 to 2020 (APC: 22; 95% CI: 11-36).

**Figure 3:**
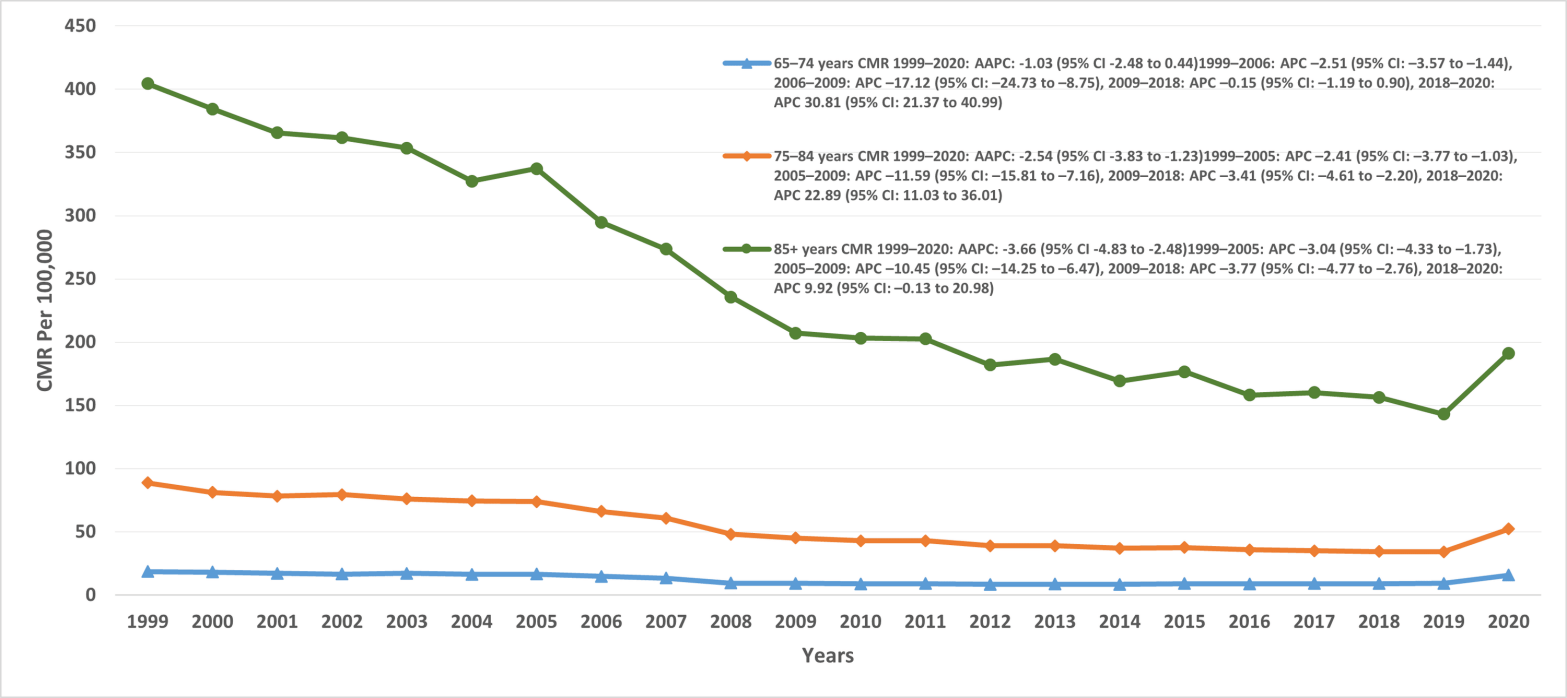
CMR trends across different age groups CMR: crude mortality rate, AAPC: average annual percentage change, APC: annual percent change.

Among individuals aged ≥85 years, the overall CMR was 235.81 (95% CI: 234.93-236.68). Mortality decreased from 1999 to 2005 (APC: -3; 95% CI: -4.3 to -1.7), continued to decline from 2005 to 2009 (APC: -10.4; 95% CI: -14.2 to -6.4), and persisted through 2018 (APC: -3.7; 95% CI: -4.7 to -2.7). Between 2018 and 2020, however, a modest uptick was observed (APC: 9.9; 95% CI: -0.13 to 20.9), though this finding may reflect statistical instability (Figure 3, Table 2).

Disparities by geographical area

A wide variation in AAMRs per 100,000 was observed across US states. The highest rates were recorded in West Virginia (AAMR: 88; 95% CI: 85.7-90.3), followed by Kentucky (AAMR: 86.4; 95% CI: 84.7-88), Oklahoma (AAMR: 82.3; 95% CI: 80.6-84), and Mississippi (AAMR: 79.3; 95% CI: 77.3-81.2). In contrast, the lowest rates were found in the District of Columbia (AAMR: 37.8; 95% CI: 34.8-40.8), Nevada (AAMR: 34; 95% CI: 32.5-35.4), Florida (AAMR: 28; 95% CI: 27.6-28.3), and Arizona (AAMR: 27.9; 95% CI: 27.1-28.6).

**Figure 4:**
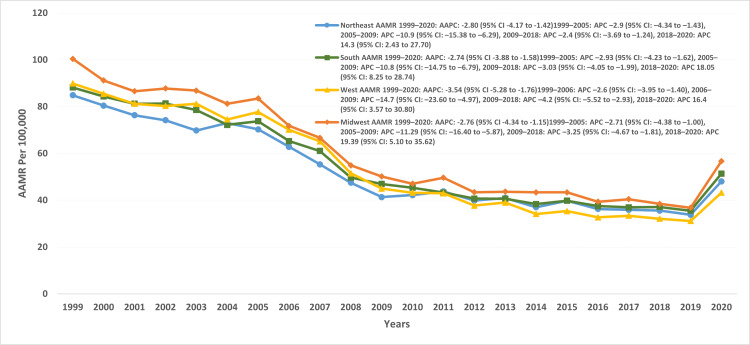
AAMR trends across different US census regions AAMR: age-adjusted mortality rate, AAPC: average annual percentage change, APC: annual percent change.

The Midwest region exhibited the highest mortality trends per 100,000 (AAMR: 100.4; 95% CI: 98.3-102.5), followed by the West (AAMR: 89.9; 95% CI: 87.6-92.2), South (AAMR: 88.1; 95% CI: 86.5-89.8), and Northeast (AAMR: 84.8; 95% CI: 82.7-86.9) (Figure 4, Table 2).

**Figure 5:**
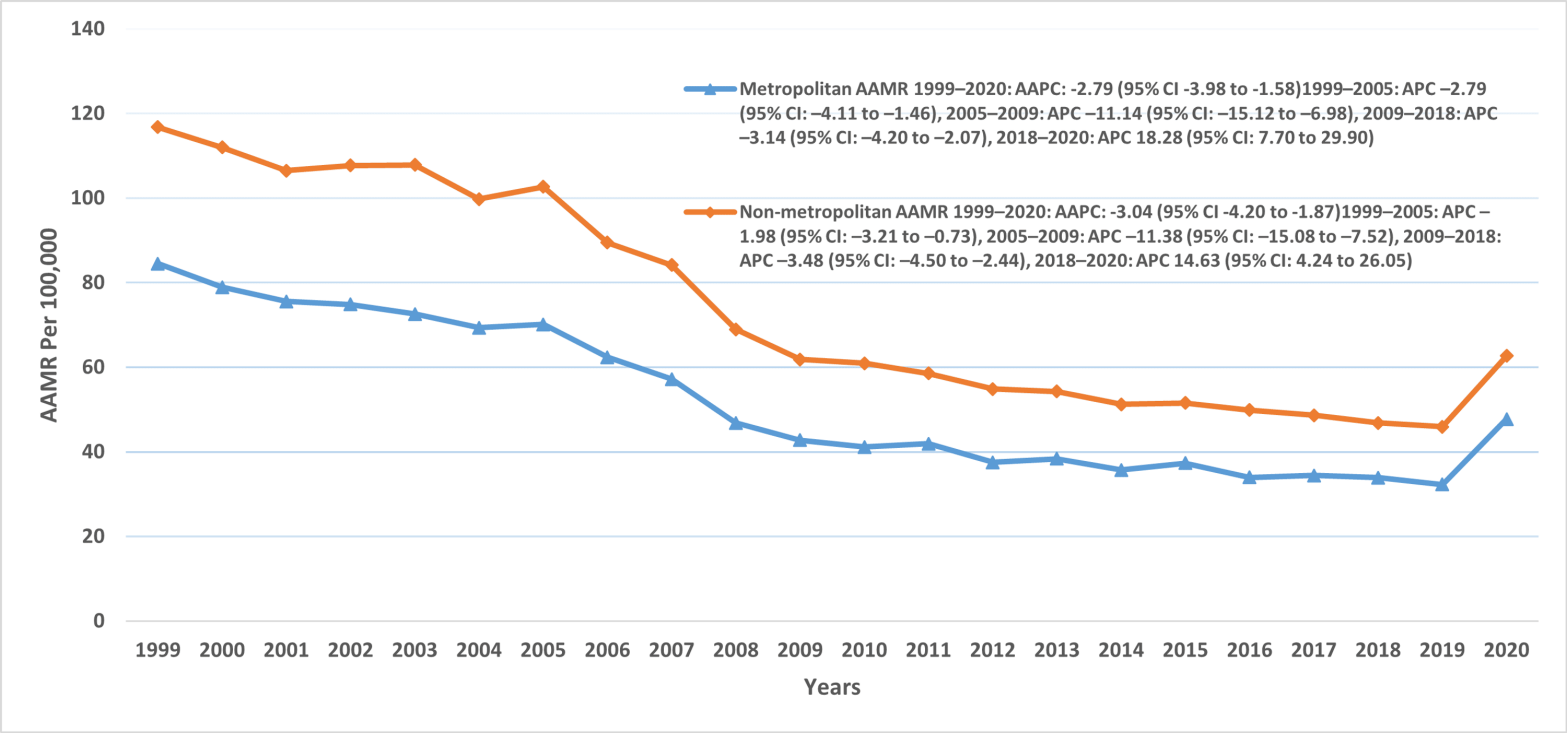
AAMR trends associated with urbanization AAMR: age-adjusted mortality rate, AAPC: average annual percentage change, APC: annual percent change.

Across urbanization levels, nonmetropolitan areas consistently demonstrated higher mortality compared with metropolitan areas. The overall AAMR per 100,000 was 72.9 (95% CI: 72.5-73.3) in nonmetropolitan areas versus 50.1 (95% CI: 49.9-50.3) in metropolitan areas. In metropolitan regions, mortality declined from 1999 to 2005 (APC: -2.79; 95% CI: -4.1 to -1.4), followed by a sharp drop between 2005 and 2009 (APC: -11.1; 95% CI: -15.1 to -6.9), and a slower decline through 2018 (APC: -3.14; 95% CI: -4.2 to -2.07). However, mortality rose markedly between 2018 and 2020 (APC: 18.2; 95% CI: 7.7-29.9). A similar trend was observed in nonmetropolitan regions, with an initial moderate decline from 1999 to 2005 (APC: -1.98; 95% CI: -3.2 to -0.7), an accelerated drop between 2005 and 2009 (APC: -11.3; 95% CI: -15.0 to -7.5), and a slower decline from 2009 to 2018 (APC: -3.4; 95% CI: -4.5 to -2.4). From 2018 to 2020, mortality in nonmetropolitan areas also increased significantly (APC: 14.6; 95% CI: 4.2-26) (Figure 5, Table 2).

## Discussion

**Figure 6:**
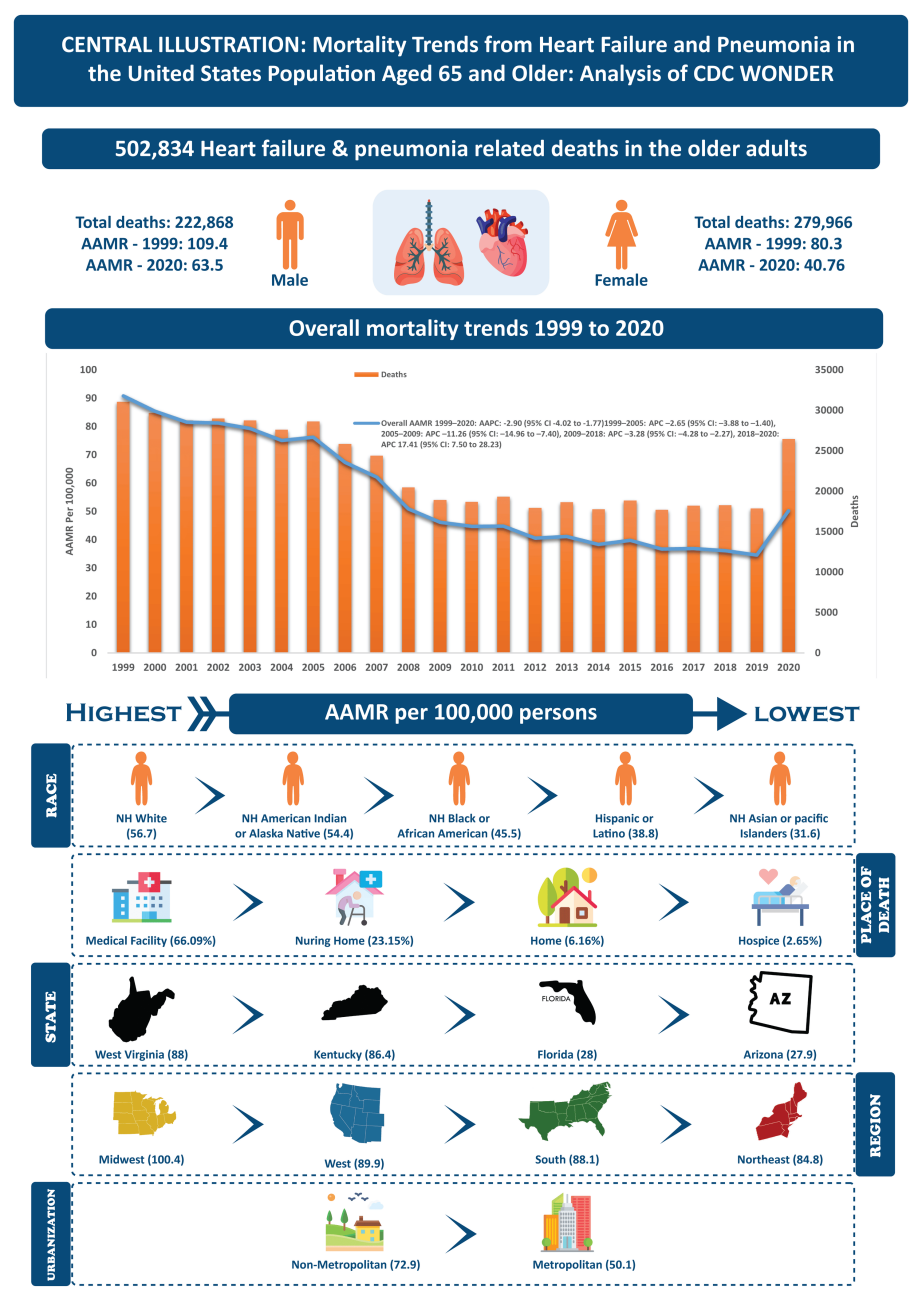
Central illustration showing mortality trends from heart failure and pneumonia among US adults aged ≥65 years NH: non-Hispanic, AAMR: age-adjusted mortality rate, AAPC: average annual percentage change, APC: annual percent change. Image Credit: This figure was created by the authors and has not been previously published.

Our 21-year study of CDC mortality statistics revealed several important trends. First, death rates declined from 1999 to 2018, then rose again between 2018 and 2020. This pattern was seen in both older men and women. Second, among adults aged 65 and older, White American adults had the highest HF- and pneumonia-related death rates compared to other racial groups. Third, rates varied widely across regions; states such as West Virginia and Kentucky had nearly double the death rates of states like Florida and Arizona. Rural areas also showed consistently higher death rates than urban areas. Regionally, mortality was highest in the Midwest, followed by the West, South, and Northeast. These findings highlight key areas for community health policy improvements (Figure 6).

This study summarizes patterns and inequities in HF- and pneumonia-related mortality among US adults aged 65 and older from 1999 to 2020. We observed a steady decline in AAMR until 2018, followed by a sharp increase from 2018 to 2020. Mortality trends varied significantly by gender, race/ethnicity, age group, geographic region, and rural-urban setting. HF mortality had been declining for over two decades, but the epidemic likely contributed to the reversal in recent years [11]. In the early 2000s, the adoption of evidence-based therapies and guideline-directed medical therapy (GDMT) improved survival and reduced HF-related complications, including pneumonia [12]. The introduction of angiotensin receptor-neprilysin inhibitors in 2015 further advanced treatment for HFrEF, improving outcomes [13]. Hospital readmission reduction initiatives also likely lowered pneumonia incidence and other comorbid complications through better transitional care [14]. Since cardiac complications are common in pneumonia and worsen both severity and cardiovascular risk, effective prevention and management strategies remain critical to reducing pneumonia-related mortality [15].

Pneumonia-related oxygen imbalance and inflammation can weaken cardiac function, worsening HF [16]. A 10-year registry study found that pneumonia patients had a significantly higher HF incidence (11.9%) compared to controls (7.4%) [17]. While the role of chronic inflammation treatment remains uncertain, vaccination may serve as a preventive strategy in HF patients [18]. Rising pneumococcal and influenza vaccination rates among older adults have likely contributed to reductions in pneumonia-related mortality [19]. Early antibiotic therapy and increased clinical awareness of pneumonia in HF patients may also have helped mitigate fatal outcomes [20]. The PARADIGM-HF trial further highlighted this vulnerability, showing that patients with HFrEF or HFpEF had double the risk of HF hospitalization and quadruple the risk of death following pneumonia [15,17].

The elevated AAMRs in HF and pneumonia may be influenced by several factors, including underlying comorbidities, the broader impact of the COVID-19 pandemic, disparities in access to care, and variations in disease management strategies. The emergence of COVID-19 in 2020 likely contributed to increased pneumonia-related mortality, particularly in HF patients, who are highly vulnerable to severe respiratory infections [21]. Global data show that since 2003, the highest cardiovascular-related deaths were recorded in 2020, coinciding with the first year of the pandemic [2]. Disruptions in HF care, such as reduced hospital visits and medication non-adherence, may have further worsened outcomes. One study reported a 43% decline in urgent cardiac admissions in March 2020, accompanied by a rise in mortality compared to previous years [22].

Our findings reveal notable racial and ethnic disparities, with older NH White adults experiencing the highest HF- and pneumonia-related AAMRs, while Asian and Pacific Islander adults had the lowest. Between 2018 and 2020, the wide confidence intervals observed in Hispanic, Asian/Pacific Islander, and American Indian/Alaska Native groups suggest statistical instability due to small sample sizes. Increasing age and multimorbidity likely contributed to higher pneumonia rates, HF readmissions, and mortality [23]. Both HF and pneumonia remain among the leading causes of hospitalization. In HF patients, 87% received beta-blockers, 75% ACE inhibitors or ARBs, and only 8% mineralocorticoid antagonists, with discontinuation of these therapies linked to increased mortality [24]. Systolic HF (HFrEF) predominantly affects men, while diastolic HF (HFpEF) is more common in women. Although women with HFpEF generally maintain better cardiac function, comorbidities substantially shape overall risk [25]. Vaccination patterns may also play a role; pneumococcal vaccination rates in a US HF registry declined from 71% in 2013 to 60% in 2016, and pneumonia incidence remained high in HF patients, particularly those with HFpEF, where it was associated with a fourfold increase in mortality [4,15,17].

Our findings highlight important geographic differences in HF- and pneumonia-related mortality among older adults, with the Midwest showing the highest AAMRs compared to other US regions. Limited access to preventive care contributes to higher HF hospitalization rates in minority populations, while socioeconomic challenges further exacerbate disparities [26]. Minority groups were also disproportionately affected by COVID-19, which may have amplified HF and pneumonia mortality [27]. Mortality was more pronounced in nonmetropolitan areas, where limited access to specialized health services often results in delayed treatment. Older adults treated in rural hospitals experience lower procedure rates and higher mortality, with critical access hospitals reporting the poorest outcomes [28]. Excess deaths from respiratory diseases have also risen more in rural areas, where diabetes, obesity, and smoking remain highly prevalent, major contributors to both HF and pneumonia [29]. Patients with chronic HF and pneumonia frequently require ICU care and benefit from intensive management, but undiagnosed HF in non-HF patients may worsen outcomes [17]. The COVID-19 pandemic further shaped these clinical trajectories, underscoring the need for tailored treatment strategies to improve quality of life and reduce disease burden [11]. Expanding telemedicine has proven valuable for HF care and is expected to remain a key tool beyond the pandemic [30].

Our findings emphasize the importance of promoting vaccination uptake among patients with HF and strengthening rural healthcare systems to improve access to preventive services, early detection, and timely management of both HF and pneumonia. The rise in mortality after 2018 may partly reflect the effects of the pandemic, underscoring the need for stronger surveillance, better linkage of mortality data with clinical datasets, and further research to assess post-pandemic mortality trends.

Limitations

This study has several limitations. First, it relies on death certificate data from the CDC WONDER database, which is subject to misclassification bias, underreporting, and inconsistencies in ICD-10 coding that may vary across regions. Because both HF and pneumonia are listed as causes of death, it is difficult to determine whether the observed increases in mortality are primarily driven by HF, pneumonia, or their combined effects. Second, as a descriptive, observational study, causality cannot be established; the findings reflect associations and temporal trends rather than direct cause-and-effect relationships. Third, the lack of individual-level data and clinical details, such as medication adherence, vaccination status, healthcare access, and hospital care, limits the ability to adjust for or assess the role of these important factors. Fourth, excluding non-resident deaths may slightly underestimate the overall mortality burden. Despite these limitations, the study provides meaningful insights into evolving patterns and disparities in HF and pneumonia mortality and highlights areas for future research.

## Conclusions

This 21-year analysis shows important trends and disparities in HF and pneumonia mortality among older US adults. Mortality declined steadily until 2018, then rose again in 2020, likely reflecting pandemic-related disruptions and heightened vulnerability in this age group. Persistent differences by sex, race, geography, and urban-rural status point to ongoing health inequities, with the Midwest and rural regions carrying the greatest burden. Early declines were probably supported by advances in evidence-based therapies and preventive measures such as vaccination. These findings underscore the need for equitable public health strategies, stronger healthcare delivery, and continued research to track post-pandemic mortality patterns and reduce preventable deaths in high-risk older adults.
